# Current practices and challenges in the management of acute bacterial skin and skin structure infections (ABSSSI): results from an Italian multicentre survey with expert discussion

**DOI:** 10.1093/jacamr/dlag119

**Published:** 2026-06-17

**Authors:** Lidia Gazzola, Matteo Augello, Silvia Cavinato, Antonio Briozzo, Federico D’Amico, Nicola Schiano Moriello, Francesco Vladimiro Segala, Luisa Frallonardo, Francesco Di Gennaro, Annalisa Saracino, Danilo Tacconi, Michele Gilio, Maria Frontuto, Giustino Parruti, Annamaria Cattelan, Giulia Marchetti

**Affiliations:** Clinic of Infectious Diseases and Tropical Medicine, San Paolo Hospital, ASST Santi Paolo e Carlo, Department of Health Sciences, University of Milan, Milan, Italy; Clinic of Infectious Diseases and Tropical Medicine, San Paolo Hospital, ASST Santi Paolo e Carlo, Department of Health Sciences, University of Milan, Milan, Italy; Infectious and Tropical Diseases Unit, Padua University Hospital, Department of Medical Sciences, University of Padua, Padua, Italy; Internal Medicine Unit, Ordine Mauriziano Hospital, Turin, Italy; Infectious Diseases Unit, ASST Grande Ospedale Metropolitano Niguarda, Milan, Italy; Clinic of Infectious Diseases, Department of Clinical Medicine and Surgery, University of Naples Federico II, Naples, Italy; Clinic of Infectious Diseases, Giovanni XXIII Hospital, Department of Biomedical Sciences and Human Oncology, University of Bari ‘Aldo Moro’, Bari, Italy; Clinic of Infectious Diseases, Giovanni XXIII Hospital, Department of Biomedical Sciences and Human Oncology, University of Bari ‘Aldo Moro’, Bari, Italy; Clinic of Infectious Diseases, Giovanni XXIII Hospital, Department of Biomedical Sciences and Human Oncology, University of Bari ‘Aldo Moro’, Bari, Italy; Clinic of Infectious Diseases, Giovanni XXIII Hospital, Department of Biomedical Sciences and Human Oncology, University of Bari ‘Aldo Moro’, Bari, Italy; Infectious Diseases Unit, San Donato Hospital, Arezzo, Italy; Infectious Diseases Unit, San Carlo Hospital, Potenza, Italy; Infectious Diseases Unit, San Carlo Hospital, Potenza, Italy; Infectious Diseases Unit, Pescara General Hospital, Pescara, Italy; Infectious and Tropical Diseases Unit, Padua University Hospital, Department of Medical Sciences, University of Padua, Padua, Italy; Clinic of Infectious Diseases and Tropical Medicine, San Paolo Hospital, ASST Santi Paolo e Carlo, Department of Health Sciences, University of Milan, Milan, Italy

## Abstract

**Background and objectives:**

Acute bacterial skin and skin structure infections (ABSSSI) are common infections associated with relevant clinical and organizational burden. Their management requires appropriate antibiotic therapy and integrated healthcare organization; however, real-world practices remain heterogeneous. The objective was to explore current clinical and organizational practices in ABSSSI management across expert Italian centres, identifying strengths, critical issues, and unmet needs through a multicentre survey with expert interpretation.

**Methods:**

A structured 107-item questionnaire was administered via e-mail to 9 Italian centres with consolidated experience in ABSSSI management. A single senior specialist provided a comprehensive response for each centre. Findings were subsequently discussed by a multidisciplinary group of key opinion leaders to contextualize results and identify priority areas for improvement.

**Results:**

Marked heterogeneity was observed across centres in organizational models and care pathways. Infectious Diseases specialists were central to management, although 24/7 availability was limited. Access to microbiology services and vulnology support varied across centres. No centre reported fully structured ABSSSI care pathways, although informal internal manuals were common. Dedicated multidisciplinary teams were present in a minority of centres. Structured communication with general practitioners and defined hospital-to-community pathways were generally lacking. Monitoring systems and dedicated databases were inconsistently implemented, with limited pharmacoeconomic evaluation.

**Conclusions:**

This multicentre survey highlights substantial variability in ABSSSI management across Italian expert centres, with key gaps in care pathways, specialist availability, and data collection. Addressing these issues may improve standardization and efficiency of care.

## Introduction

Acute bacterial skin and skin structure infections (ABSSSI) are bacterial infections of the skin and associated soft tissues encompassing cellulitis/erysipelas, wound infections, and major cutaneous abscesses.^1^ Common bacterial pathogens causing ABSSSI are *Streptococcus pyogenes* and *Staphylococcus aureus*, including methicillin-resistant *S. aureus* (MRSA).^2^ In the past decade, there has been a dramatic increase in the incidence of community-acquired skin infections, most of which are caused by MRSA. In Europe, despite high variability in prevalence, MRSA isolation has not only reached but even exceeded 25% of ABSSSI cases, especially in areas where antimicrobial resistance is a significant issue.^3^

The management of ABSSSI extends beyond the selection of appropriate antimicrobial therapy and requires a coordinated multidisciplinary and organizational approach.^4^ Early clinical recognition, accurate microbiological assessment, timely Infectious Diseases consultation, appropriate surgical and wound care support, antimicrobial stewardship, and continuity of care all contribute to optimal patient management. In addition, the increasing complexity of healthcare systems has highlighted the importance of structured clinical pathways capable of integrating inpatient and outpatient care, facilitating early discharge strategies, and optimizing healthcare resource utilization. In this context, long-acting antibiotics have emerged as a potential therapeutic option in selected patients, particularly within outpatient management programmes and strategies aimed at reducing hospitalization length and improving continuity of care.^5,6^

Despite the clinical and organizational relevance of ABSSSI, significant variability still exists among centres in terms of specialist availability, microbiology support, integrated care pathways, multidisciplinary collaboration, monitoring systems, and communication with territorial healthcare services.^4^ Understanding current practices and identifying organizational gaps may help define potential areas for improvement and support the development of more standardized and efficient management strategies.

The aim of the present study was therefore to explore current clinical and organizational practices related to the management of ABSSSI across expert Italian centres, in order to map existing organizational models, identify strengths and critical issues, and highlight unmet needs, ultimately contributing to the definition of priority areas for future improvement in ABSSSI management.

## Methods

A multidisciplinary working group, consisting of 20 key opinion leaders (KOLs)—including Infectious Diseases specialists, residents, and nurses—from nine Italian centres, held a series of periodic meetings to explore various aspects related to the treatment of ABSSSI and complicated infections. Centres were selected based on their consolidated clinical experience in the management of ABSSSI, as the primary objective of the study was to explore and map current organizational models, management approaches, and unmet needs in centres with established expertise in this field. Among the nine hospitals included in the study, seven are tertiary care hospitals, and three of them have a capacity of more than 1000 beds (Table S1, available as Supplementary data at *JAC-AMR* Online).

During an initial virtual meeting, KOLs identified seven key topics: context, organization, clinical assessment, ABSSSI, and complicated infection indicators, staff training, quality, and research activities. Then, an independent methodologist provided a structured survey in which each of previously discussed topics was dissected into 107 open- and close-ended questions (Table S2). The survey was administered as an Excel questionnaire distributed by e-mail. At each participating centre, a single designated respondent—typically the Head of the Infectious Diseases Unit or an equivalent senior specialist—provided a consolidated response reflecting the organizational and clinical practices of the centre. This approach was intentionally adopted to ensure consistency and reliability of the information reported, as the selected respondents have a comprehensive overview of local pathways, antimicrobial stewardship practices, and organizational structures related to ABSSSI management. All nine centres that were invited completed the survey, yielding a response rate of 100%.

Subsequently, the methodologist analysed the resulting data from all nine centres. Given the exploratory nature of the study, data were analysed using descriptive statistics, primarily proportions. No formal sample size calculation was performed, as the study was not designed to test a hypothesis but to map practices in a purposively selected sample of expert centres. Missing data were minimized by design: in cases where responses were incomplete or unclear, follow-up contacts were conducted to retrieve the missing information. As a result, no relevant missing data were present in the final dataset. Due to the study design—one consolidated response per centre—a formal analysis of non-response bias was not applicable.

The resulting data were discussed in subsequent meetings. Finally, the expert panel participated in preparation, discussion, and revision of the final document.

## Results

### Clinical and laboratory assessment criteria for ABSSSI: consensus, risk factors, and comorbidity evaluation

From a clinical perspective, all centres agree on assessing *rubor*, *tumor*, *calor*, fever, lymphadenopathy, and skin crepitation as signs of ABSSSI. Regarding blood tests, 89% of centres suggests evaluating one or more of the following tests, including white blood cells, C-reactive protein, liver function, coagulation, and the Laboratory Risk Indicator for Necrotizing Fasciitis (LRINEC) in cases of suspected necrotizing fasciitis, along with lactate dehydrogenase (LDH), bilirubin, procalcitonin, monocyte distribution width (MDW), creatinine, and electrolytes during the initial assessment to obtain a comprehensive clinical picture.

Additionally, during the assessment phase, all centres further investigate risk factors, previous episodes of ABSSSI and/or complicated infections, recent surgical interventions, lymphatic or venous stasis, immunosuppression, pre-existing skin disorders, skin trauma and/or animal bites, diabetes mellitus, obesity, and intravenous substance use. Only a minority of centres routinely use comorbidity assessment scales, such as the Charlson Comorbidity Index (33%) or the Cumulative Illness Rating Scale (10%). These indices are more commonly used in research, which is conducted in 67% of the centres involved in the study.

### ABSSSI and complicated infections management: specialist availability, service gaps, coordination issues, and the need for improved integration

**Figure 1.**
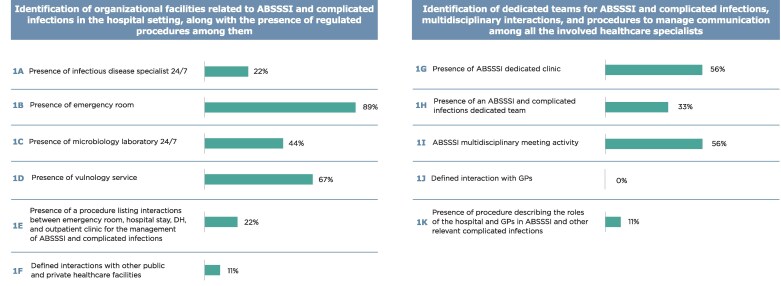
Identification of organizational facilities related to ABSSSI and complicated infections in the hospital setting, along with the presence of regulated procedures among them (a–f). Identification of dedicated teams for ABSSSI and complicated infections, multidisciplinary interactions, and procedures to manage communication among all the involved healthcare specialists (g–k). Percentages represent the proportion of affirmative responses out of the nine involved hospitals. Legend: *ABSSSI*, acute bacterial skin and skin structure infections; *DH*, day hospital; *GP*, general practitioner.

The key figure in managing patients with ABSSSI is the Infectious Diseases specialist. However, most hospitals do not have this specialist available 24/7 (Figure 1a). Several of them reported that not all ABSSSI patients are managed by an Infectious Diseases specialist. Often, patients are examined by an Emergency Room physician, available in nearly all centres (Figure 1b), or, if hospitalized for other reasons, by an Internal Medicine specialist. These professionals may sometimes request an Infectious Diseases consultation but may also manage the patient entirely.

Isolating and identifying bacterial pathogens allows for the initiation of targeted antibiotic therapy. Therefore, the availability of a 24/7 microbiology service, capable of reducing turnaround times, can shorten the duration of empirical therapy in favour of targeted therapy. The availability of a round-the-clock microbiology service is limited to a few centres, but it is recognized as an added value in terms of efficiency in the care pathway (Figure 1c).

Another important service in ABSSSI treatment is vulnology (wound care), which is available in six centres (67%) (Figure 1d). Nurses in this service play a pivotal role in the assessment and diagnosis phase by characterizing the wound nature, including its depth, extent, and type, which aids in choosing the appropriate treatment. Additionally, proper vulnology techniques are essential to promote healing and prevent complications. This includes cleaning the wound, debriding necrotic tissue, and applying appropriate dressings, thus helping to prevent complications such as secondary infections, abscess formation, or systemic spread of infection.

Patient management can take place in all the previously mentioned settings or in dedicated ABSSSI clinics, which are present in more than half of the centres. However, only two centres reported having specific procedures outlining the proper interaction between the emergency room, inpatient care, day hospital, and outpatient clinic. Clinicians stated that while these procedures could improve the patient’s journey, they are not essential for managing these infections. Furthermore, since these are not chronic conditions, establishing dedicated channels with other public or private hospital facilities is deemed unnecessary (Figure 1e–g).

Only three centres (33%) have a multidisciplinary team dedicated to ABSSSI, while the other centres discuss complex patients in multidisciplinary meetings focused on a broader range of conditions (Figure 1h,i). The specialists involved usually include a diabetologist, vascular surgeon, nurses specialized in vulnology, and a pharmacist.

One key element lacking across all centres is the establishment of a well-organized network connecting with general practitioners (Figure 1j–k). This issue is particularly evident in hospital organizations that lack continuity of care with general practitioners. Some centres report that these practitioners are often not adequately trained in identifying this pathology and that it would be beneficial to develop tools, such as a dedicated e-mail, to communicate with Infectious Diseases specialists. This tool would facilitate more appropriate and timely patient referrals.

### Dedicated ABSSSI pathways: current practices, training, and protocols

**Figure 2.**
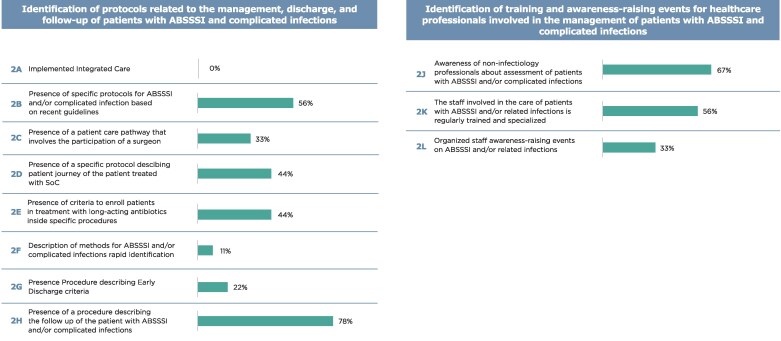
Identification of protocols related to the management, discharge, and follow-up of patients with ABSSSI and complicated infections (a–h). Identification of training and awareness-raising events for healthcare professionals involved in the management of patients with ABSSSI and complicated infections (j–l). Percentages represent the proportion of affirmative responses out of the nine involved hospitals. Legend: *ABSSSI*, acute bacterial skin and skin structure infections; *SoC*: standard of care.

Although patients with these conditions may be discussed in multidisciplinary meetings and various specialists can be involved in their treatment, no centre has reported the presence of a dedicated care pathway for ABSSSI (Figure 2a). The absence of such dedicated procedures leads some centres to manage the entire process through consultations or messaging systems that are not integrated within the hospital structure. All nine centres believe that developing an integrated care pathway would optimize the patient management. However, the current shortage of qualified personnel to create such pathways leads centres to prioritize other, more complex pathways. Despite the absence of written protocols (i.e. formally endorsed institutional documents), most specialists have developed dedicated manuals for ABSSSI and other conditions (informal, non-institutionally endorsed documents developed by individual specialists), which are updated periodically and are accessible to all other specialists within the institution (Figure 2b). This helps standardize the management and treatment of patients. Three centres (33%) also detailed a procedure describing the surgeon’s involvement in the patient care pathway (Figure 2c).

Additionally, four centres (44%) have described treatment criteria using standard-of-care therapies and long-acting antibiotics in specific procedures. However, only a minority of centres have detailed specific protocols for the rapid identification of ABSSSI and criteria for early discharge or patient follow-up (Figure 2d–h). Despite this, specialists from all centres agree on using the same criteria to facilitate early patient discharge, even though many centres have not documented these criteria in a written procedure. The criteria for early discharge include a stable clinical condition of the lesion, absence of fever for at least 24 h, a normal white blood cell count, absence of signs of systemic inflammatory response syndrome (SIRS), and no other reasons for hospitalization apart from treating the skin infection.

Although the procedures described earlier are not detailed in a document, most centres (67%) believe that non-infectious disease specialists who occasionally manage ABSSSI patients are adequately trained and knowledgeable about these conditions (Figure 2j). Additionally, three out of nine centres (33%) organize dedicated training events to educate all specialists involved in managing patients affected by these infections (Figure 2k–l).

### Monitoring and data management of ABSSSI and complicated infections across centres

Regarding the monitoring of these infections, all centres closely use structured tools to assess the incidence of healthcare-associated infections and MRSA infections (89% and 67%, respectively). Less than half of the centres (44%) have a dedicated database for ABSSSI or record incidence and prevalence. However, there is a robust monitoring system for all infections, from which data extraction regarding the comorbidities and risk factors of patients with ABSSSI and complicated infections is possible (78%). Most centres also record treatment outcome data (56%).

From a pharmacoeconomic standpoint, however, only pharmacological therapy costs and prevented hospitalization are considered for patients treated with long-acting antibiotics. Regarding this type of data, clinicians explained that their hospital administrations currently require monitoring pharmacological therapy costs, although not stratified by specific pathology. As for organizational costs, particularly those related to hospital stays, strict monitoring is not required. On the patient side, only one centre reported monitoring customer satisfaction concerning ABSSSI and complicated infections.

## Discussion

This study provides a comprehensive overview of the current practices and challenges in managing ABSSSI across multiple centres in Italy, revealing some variability in specialist availability, service integration, and the adoption of structured care pathways.

In face of a strong consensus among clinicians regarding clinical and laboratory assessment criteria for ABSSSI, our findings reveal a unanimous recognition of the need for integrated care pathways among the surveyed centres, yet none have implemented such a system. This discrepancy between recognized need and actual practice is largely attributed to the shortage of qualified personnel, which forces centres to prioritize more complex pathways. Nevertheless, the development of dedicated manuals and protocols, albeit informal, indicates a positive step towards standardizing care.

The microbiology service is a valuable support in managing ABSSSI, especially the more complicated ones. A limited availability of the service may delay response times and the approach through targeted therapy.

While dedicated ABSSSI clinics exist in several centres, only a minority have established clear procedural guidelines to streamline patient care across these settings. The lack of multidisciplinary teams specifically dedicated to ABSSSI complicates patient management, as complex cases often require input from various specialists other than Infectious Diseases specialists, including diabetologists, vascular surgeons, and pharmacists.

In the opinion of the working group, an integrated care pathway for ABSSSI should extend beyond the isolated availability of protocols or multidisciplinary expertise and instead provide a coordinated organizational framework across all phases of patient management.^4^ Key elements would include (i) standardized triage and diagnostic criteria in the emergency department; (ii) timely Infectious Diseases consultations and microbiology support; (iii) access to vulnology services and surgical evaluation when required; (iv) shared antimicrobial stewardship strategies;^7^ (v) formalized criteria for hospitalization and early discharge; (vi) outpatient or day hospital follow-up pathways;^8^ and (vii) structured communication with general practitioners to ensure continuity of care after discharge. Such a model could facilitate more appropriate patient allocation, reduce unnecessary hospitalizations, promote earlier transition to outpatient management, and improve consistency in clinical decision-making across different hospital settings. Similar multidisciplinary and transition-of-care approaches have previously been proposed to optimize ABSSSI management and reduce unnecessary hospitalizations and inappropriate antimicrobial use.^4,6,7^ In this context, long-acting antibiotics may represent an important component of integrated care pathways by supporting early discharge strategies and facilitating outpatient management in selected patients.^6^

The study also highlights gaps in monitoring and data management of ABSSSI across the centres. The lack of a dedicated monitoring system hinders the ability to accurately assess the incidence, prevalence, and treatment outcomes of these infections. This gap also limits cost-effectiveness and pharmacoeconomic studies, which are essential for evaluating the best care strategies in both clinical and managerial terms. When comparing long-acting antibiotics with the standard of care, it is crucial to evaluate savings in terms of effectiveness, practicality, and early discharge, as well as to highlight the hidden costs associated with hospitalization, from both a clinical and economic standpoint. Given the growing importance of data-driven decision-making, these deficiencies represent critical areas for improvement in the Italian healthcare system.

Several limitations of this study should be acknowledged. First, only nine centres participated, and their selection was based on clinical expertise rather than random sampling; therefore, representativeness and generalizability of the findings to all Italian or European centres managing ABSSSI may be limited. The results should be interpreted as reflecting practices at high-volume, experienced centres rather than as a nationally representative picture. Second, each centre provided a single consolidated response through a designated senior respondent, which, while ensuring consistency, may not fully capture the heterogeneity of practices within each institution. Third, no formal consensus methodology (e.g. Delphi process) was applied; conclusions therefore reflect structured expert discussion rather than a validated consensus.

In conclusion, this study identifies several opportunities for improvement. Addressing these gaps through enhanced specialist availability, the establishment of integrated care pathways, and improved data management could lead to more consistent and effective patient care, ultimately improving outcomes for those affected by these serious infections.

## Supplementary Material

dlag119_Supplementary_Data

## Supplementary data

Tables S1 and S2 are available as Supplementary data at *JAC-AMR* Online.

## References

[1] Golan Y . Current treatment options for acute skin and skin-structure infections. Clin Infect Dis 2019; 68: S206–12. 10.1093/cid/ciz004 30957166 PMC6451992

[2] Russo A, Concia E, Cristini F et al Current and future trends in antibiotic therapy of acute bacterial skin and skin-structure infections. Clin Microbiol Infect 2016; 22(Suppl 2): S27–36. 10.1016/S1198-743X(16)30095-7 27125562

[3] Esposito S, Noviello S, Leone S. Epidemiology and microbiology of skin and soft tissue infections. Curr Opin Infect Dis 2016; 29: 109–15. 10.1097/QCO.0000000000000239 26779772

[4] Pollack CV, Amin A, Ford WT et al Acute bacterial skin and skin structure infections (ABSSSI): practice guidelines for management and care transitions in the emergency department and hospital. J Emerg Med 2015; 48: 508–19. 10.1016/j.jemermed.2014.12.001 25605319

[5] Tran TT, Gomez Villegas S, Aitken SL et al New perspectives on antimicrobial agents: long-acting lipoglycopeptides. Antimicrob Agents Chemother 2022; 66: e0261420. 10.1128/aac.02614-20 35475634 PMC9211417

[6] Oliva A, Carbonara S, Cianci V et al Direct or early discharge of acute bacterial skin and skin structure infection patients from the emergency department/unit: place in therapy of dalbavancin. Expert Rev Anti Infect Ther 2023; 21: 703–21. 10.1080/14787210.2023.2214727 37227028

[7] Soriano A, Stefani S, Pletz MW et al Antimicrobial stewardship in patients with acute bacterial skin and skin-structure infections: an international Delphi consensus. J Glob Antimicrob Resist 2020; 22: 296–301. 10.1016/j.jgar.2020.02.002 32068092

[8] Mahoney MV, Childs-Kean LM, Khan P et al Recent updates in antimicrobial stewardship in outpatient parenteral antimicrobial therapy. Curr Infect Dis Rep 2021; 23: 24. 10.1007/s11908-021-00766-x 34776793 PMC8577634

