# Supplementary material for: Current practices and challenges in the management of acute bacterial skin and skin structure infections (ABSSSI): results from an Italian multicentre survey with expert discussion
Source: JAC Antimicrob Resist. 2026 Jun 17;8(3):dlag119. doi: 10.1093/jacamr/dlag119 (PMC13273421; doi:10.1093/jacamr/dlag119)
Supplement: dlag119_Supplementary_Data [file dlag119_supplementary_data.pdf]

Supplementary material

Supplementary Table 1

Hospital	Bed capacity	Emergency Care Level	Hospital Type
Santi Paolo Hospital, ASST Santi Paolo e Carlo, Milan	1100	Level I	University-affiliated
Padua University Hospital, Padua	1700	Level II	University-affiliated; tertiary care hospital
Mauriziano Hospital, Turin	470	Level II	Teaching hospital; tertiary care hospital
Niguarda Metropolitan Hospital, Milan	1167	Level II	Teaching hospital; tertiary care hospital
Federico II University Hospital, Naples	860	Level I	University-affiliated; tertiary care hospital
Giovanni XXIII Children's Hospital, Bari	218	Level II	University-affiliated; tertiary care hospital
San Donato Hospital, Arezzo	400	Level I	Teaching hospital
San Carlo Hospital, Potenza	750	Level II	University-affiliated; tertiary care hospital
Pescara General Hospital	550	Level I	Teaching hospital; tertiary care hospital

Supplementary Table 2

The Survey on ABSSSI and Complicated Infections, submitted to clinicians from nine Italian Infectious Disease Units, to map best practices and virtuous governance models.

Macroarea	Requirements
Context	Is an Infectious Diseases Unit (IDU) present?
Context	Is the Infectious Diseases Unit part of an IRCCS?
Context	Does the medical team of the Infectious Diseases Unit operate within a context strictly related to the academic world?
Context	Has The center an Emergency Department?
Context	If yes, is the infectious disease specialist physically present on-site 24/7?
Context	If yes, is the infectious disease specialist available on call 24/7?
Context	If yes, is the infectious disease specialist available remotely at specific hours (e.g., night-time)?
Context	Has the center non-infectious disease specialists - possibly recruited by the Infection Control Committee (CIO) - for administering long-acting antibiotics?
Context	If yes, what are the specializations of these professionals?
Context	Is there a dedicated team for infections and/or ABSSSI within the facility?
Context	If yes, list all involved professionals?
Context	Is there an outpatient service dedicated to ABSSSI and/or complicated infections within the Infectious Diseases outpatient clinics?
Context	If yes, how is booking for this outpatient service done?
Context	Are there agreements with other public and/or private healthcare facilities for management of ABSSSI and/or complicated infections?

Context	Are there agreements with other public and/or private social health facilities for management of ABSSSI and/or complicated infections?
Context	Are there agreements with general practitioners' outpatient clinics for management of ABSSSI and/or complicated infections?
Context	How many years of experience do the infectious disease specialists providing consultation have?
Context	What type of experience do the infectious disease specialists providing consultation have?
Organization	Does a Diagnostic-Therapeutic Care Pathway (PTDA) for managing ABSSSI and/or complicated infections exist?
Organization	Do well-defined treatment protocols based on updated guidelines exist?
Organization	Are patient enrollment criteria for treatment with long-acting antibiotics clear and included in a specific procedure?
Organization	There is a procedure describing interactions between the facility and territory for managing ABSSSI and/or complicated infections?
Organization	Is there a procedure describing internal interactions among the Emergency Department, wards, day hospitals, and outpatient clinics for managing ABSSSI and/or complicated infections?
Organization	Is there a rapid identification method for ABSSSI and/or complicated infections described in a specific procedure?
Organization	Is there a microbiology service (24/7) capable of analyzing tissue/fluid samples from patients with ABSSSI and/or complicated infections?
Organization	Is there a radiology service (24/7) capable of promptly assessing the extent of infection and possible involvement of deep tissues?
Organization	Is there a pharmacy service (24/7) capable of supporting wards and/or services in case of long-acting antibiotics prescription?
Organization	If no, on which days and during which hours is the pharmacy service active?
Organization	Is there a wound care service?
Organization	If yes, on which days and during which hours is this service available?
Organization	If yes, how is managed the access to the wound care service (e.g., booking through CUP, booking only by hospital specialist)?
Organization	Is there a follow-up program for patients with ABSSSI?
Organization	If yes, is the patient with ABSSSI and/or complicated infections reassessed 24-72 hours after diagnosis and/or start of antibiotic treatment?
Organization	If yes, is the patient with ABSSSI and/or complicated infections reassessed 7 days after diagnosis and/or start of antibiotic treatment?
Organization	If yes, is the patient with ABSSSI and/or complicated infections reassessed at the end of antibiotic therapy?
Organization	Are multidisciplinary meetings organized to discuss complex cases of patients with ABSSSI and/or complicated infections?
Organization	Are electronic health records implemented at the center?
Organization	Are Telemedicine services available for patients with ABSSSI and/or complicated infections?
Organization	Are costs related to ABSSSI and/or associated infections divided among individual wards/cost centers?
Assessment	Is there a system to stratify the risk profile of each patient?
Assessment	Does the clinical-care complexity of patients include assessment of various factors: risk factors, previous episodes of ABSSSI and/or complicated infections, recent surgery, lymphatic or venous stasis, immunosuppression, pre-existing skin disorders, skin trauma and/or animal bites, diabetes mellitus, obesity, intravenous substance use? Are all these aspects always assessed for each patient?
Assessment	If no, which of the above aspects are assessed?
Assessment	If other aspects are assessed, please specify
Assessment	Does clinical evaluation include collection of signs/symptoms: redness/swelling/heat, fever, lymphadenopathy, skin crepitus?<br>Are all these aspects always assessed for each patient?
Assessment	If no, which of these signs/symptoms are assessed?
Assessment	If other aspects are assessed, please specify

Assessment	Regarding blood tests during initial assessment of patients with ABSSSI and/or complicated infections, does the infectious disease specialist evaluate white blood cells and CRP? Are there other laboratory parameters assessed in managing a patient with ABSSSI?
Assessment	If yes, specify other parameters evaluated
Assessment	Are comorbidities of patients with ABSSSI and/or complicated infections assessed using specific tools such as the Charlson Comorbidity Index (CCI)?
Assessment	Are comorbidities of patients with ABSSSI and/or complicated infections assessed using specific tools such as the Cumulative Illness Rating Scale (CIRS)?
Assessment	If other tools are used to assess comorbidities, specify which
Assessment	In case of treatment ABSSSI and/or complicated infections with "Standard of care", is the patient pathway described in a specific protocol?
Assessment	If yes, is any switch to long-acting antibiotic therapy recorded?
Assessment	If yes, are reasons for switching to long-acting antibiotic therapy recorded?
Assessment	Before administration of long-acting antibiotic therapy, the nurse evaluates the patient's venous access
Assessment	Criteria regarding dosing of long-acting antibiotics are well defined and included in specific procedure
Assessment	Criteria regarding reconstitution and dilution of long-acting antibiotics are well defined and included in specific procedure
Assessment	Administration times of long-acting antibiotics are clear and included in specific procedure
Assessment	Storage criteria for long-acting antibiotics are clear to involved staff and included in specific procedure
Assessment	There is a digital tracking method for long-acting antibiotics
Assessment	Criteria related to Early Discharge are defined and included in specific procedure
Assessment	If yes, what are they?
Assessment	If yes, what are the contraindications to early discharge?
Assessment	Non-infectious disease professionals are aware of patient assessment criteria for ABSSSI and/or complicated infections
Assessment	There is a care pathway for patients with ABSSSI and/or complicated infections that involves the surgeon
Context Indicators	Are incidence and prevalence of infections (excluding ABSSSI and/or complicated infections) constantly monitored?
Context Indicators	If yes, is a periodic report shared with all staff?
Context Indicators	The center closely monitors healthcare-associated infections
Context Indicators	If yes, is a periodic report shared with all staff?
Context Indicators	The center closely monitors methicillin-resistant strains (MRSA)
Context Indicators	If yes, is a periodic report shared with all staff?
Context Indicators	Infection severity (excluding ABSSSI and/or complicated infections) is recorded in a structured manner
Context Indicators	If yes, how? (Open question, insert answer in column D)
Context Indicators	Use of specific antibiotics conforming to guidelines is monitored
Context Indicators	Treatment costs for patients with infections (excluding ABSSSI and/or complicated infections) are periodically monitored
ABSSSI and Complicated Infection Indicators	There is a database collecting data on ABSSSI and complicated infections
ABSSSI and Complicated Infection Indicators	Incidence and prevalence of ABSSSI and complicated infections are constantly monitored
ABSSSI and Complicated Infection Indicators	If yes, is a periodic report provided to staff?
ABSSSI and Complicated Infection Indicators	Comorbidities of patients with ABSSSI and/or complicated infections are recorded and monitored

ABSSSI and Complicated Infection Indicators	Risk factors of patients with ABSSSI and/or complicated infections are recorded and monitored
ABSSSI and Complicated Infection Indicators	If yes, how? (Open question, insert answer in column D)
ABSSSI and Complicated Infection Indicators	Use of specific antibiotics for ABSSSI and/or complicated infections conforming to guidelines is monitored
ABSSSI and Complicated Infection Indicators	Volume of invasive procedures and re-interventions in patients with ABSSSI and/or complicated infections is monitored
ABSSSI and Complicated Infection Indicators	Average length of stay of patients with ABSSSI and/or complicated infections treated with long-acting antibiotics is monitored
ABSSSI and Complicated Infection Indicators	Outcome of care for patients with ABSSSI and/or complicated infections is monitored and recorded
ABSSSI and Complicated Infection Indicators	Treatment costs for patients with ABSSSI and/or complicated infections are periodically monitored
ABSSSI and Complicated Infection Indicators	Customer satisfaction: patient satisfaction with received care is monitored and patients are asked to identify any areas for improvement
Staff Training	The center ensures that staff involved in managing patients with ABSSSI and/or related infections are adequately trained and specialized
Staff Training	If yes, training involves senior physicians
Staff Training	If yes, training involves members of the Hospital Infection Committee
Staff Training	If yes, training involves professional nurses
Staff Training	If yes, training involves radiologists and/or radiology technicians
Staff Training	If yes, training involves laboratory technicians
Staff Training	If yes, training involves pharmacists
Staff Training	If yes, training involves support staff
Staff Training	If yes, training involves staff responsible for hospitality activities in wards/services
Staff Training	Healthcare personnel involved in care of ABSSSI and/or complicated infections receive continuous training on ABSSSI and/or complicated infections
Staff Training	If yes, periodic courses are organized by the facility
Staff Training	If yes, what type of courses are organized by the facility? (Open question, insert answer in column D)
Staff Training	If no, do professionals train autonomously?
Staff Training	If no, do you think training courses would be useful?
Staff Training	If no, would, the involved staff, be willing to participate in training courses on ABSSSI and/or complicated infections?
Staff Training	If no, what type of training course would be most effective for staff involved (e.g., e-learning, onsite lectures, etc.)? (Open question, insert answer in column D)
Staff Training	Awareness events for staff on ABSSSI and/or related infections are currently organized at the center
Quality	There is a patient education program for those affected by ABSSSI and/or complicated infections aimed at preventing complications
Quality	If yes, how is patient education conducted? (Open question, insert answer in column D)
Quality	Periodic internal audits are conducted within the center to monitor the clinical-care pathway of patients with ABSSSI and/or complicated infections
Research Activities	The center has promoted research in the field of ABSSSI and/or complicated infections in the past year
